# Optimizing Cardiovascular Care in Aging Populations: A Comprehensive Review of Geriatric Cardiology

**DOI:** 10.7759/cureus.87992

**Published:** 2025-07-15

**Authors:** Shreya Agarwal, Ifeoma N Ozor, Siri Chithanuru, Enitan O Odumosu, Olatunji E Fadiora, Glory Ikwan, Gopichand Bhanavath, Maaz Siddiqui, Razia Sultana, Sreedharan Murugesan, Esther O Adebambi, Ramsha Ali

**Affiliations:** 1 Internal Medicine, JSS Medical College, Mysore, IND; 2 Family Medicine, University of Niš, Niš, SRB; 3 Internal Medicine, Mamata Medical College, Khammam, IND; 4 Internal Medicine, Caucasus International University, Tbilisi, GEO; 5 Internal Medicine, Windsor University School of Medicine, Bassettere, KNA; 6 Internal Medicine, New Vision University, Tbilisi, GEO; 7 Cardiac Anesthesiology, Fortis Memorial Research Institute, Gurgaon, IND; 8 Psychology, Virginia Tech, Virginia, USA; 9 Internal Medicine, American University of Antigua, Coolidge, ATG; 10 Internal Medicine, Anwer Khan Modern Medical College, Dhaka, BGD; 11 Family Medicine, Coimbatore Medical College Hospital, Coimbatore, IND; 12 Internal Medicine, University of Limerick School of Medicine, Limerick, IRL; 13 Medicine and Surgery, Peoples University of Medical & Health Sciences for Women, Hyd, PAK

**Keywords:** comprehensive geriatric assessment, geriatric cardiology, multidisciplinary treatments, multimorbidity management, polypharmacy

## Abstract

A narrative review was conducted by searching recent literature in databases like Cochrane and PubMed, using keywords such as “older adults”, “cardiovascular disease”, “frailty”, and “polypharmacy”. The identified literature included cohort studies, scientific statements, systematic reviews, meta-analyses, and narrative reviews from the last 15 years focusing on comprehensive care and recent evidence-based updates in geriatric cardiology. Inclusion criteria of the aging population and the elderly with cardiovascular conditions have been applied. The review excluded articles on pregnant women and children, studies on animals, and articles not written in English. A thematic synthesis was conducted to identify key challenges and strategies in geriatric cardiovascular care, focusing on multimorbidity, cognitive impairment, and integrative care models. Our narrative review documents definitions, findings, recent updates and practical approaches that can be implemented in geriatric practice addressing diagnostic and therapeutic challenges in geriatric cardiology; disadvantages of traditional-disease focused models in elderly; frailty, multimorbidity, polypharmacy and overcoming them; health inequities and disparities in cardiogeriatric populations; and finally about some of the areas needing future researches and policies. The global rise in the aging population has contributed to cardiovascular diseases (CVDs) becoming a dominant health concern among older adults who often present with a range of coexisting conditions. These geriatric syndromes complicate standard cardiovascular care, making collaborative multidisciplinary and patient-centered strategies essential to improve health outcomes and functional independence. This narrative review is aimed at exploring the efficacy of integrated, evidence-based multidisciplinary cardiovascular care models on improving clinical outcomes and quality of life (QoL) among geriatric adults. The objective evaluates and compares the impact of these models versus traditional practices, while also exploring key challenges and recent innovations in cardiovascular care for aging populations.

## Introduction and background

As the global population continues to age rapidly, the current populations of 700 million aged 65 and older are projected to double by 2050 and increase the burden of chronic diseases in the coming decades [1]. Cardiovascular diseases (CVDs) remain the leading cause of death in this demographic, yet older adults present with clinical challenges such as multimorbidity, frailty, cognitive impairment, and polypharmacy, all of which complicate standard cardiovascular care [2,3]. The complex interplay between these factors underscores the need for a specialized, integrative approach to geriatric cardiovascular care. This brings forth the central question: given the projected growth of the elderly population and the associated rise in CVDs, what evidence-based strategies and integrative care models can be implemented to mitigate cardiovascular risk and enhance the quality of life (QoL)?

By 2050, the aging population is predicted to increase by more than double to an unprecedented 1.5 billion. It is a common misconception that geriatric care can be managed similarly to general adult care. However, this review highlights the shortcomings of such an approach and emphasizes the necessity of a multidisciplinary model that accounts for the intricate relationship between CVD and age-related conditions [4,5]. Conventional treatment protocols often fail to address the nonlinear progression of comorbidities such as diabetes, hypertension, and chronic kidney disease in older adults. These conditions, when coupled with age-related physiological changes like diminished cardiac function and increased vascular stiffness, demand a more patient-centred strategy to achieve meaningful outcomes and address issues like polypharmacy. In addition, elderly patients undergoing chemotherapy with agents such as anthracycline and trastuzumab experience elevated cardiovascular risks as side effects. Arterial thromboembolism risk of 3.8% has been observed on treatment with bevacizumab with chemotherapy (vs. 1.7% risk with chemotherapy alone). This underscores the pressing need for more targeted clinical trials to fill existing knowledge gaps [6]. This aspect of geriatric cardiology is critical yet often underexplored.

Geriatric care extends beyond the treatment of diseases. We need to focus on the integration of cardiac rehabilitation and palliative care as well. While cardiac rehabilitation aims to restore cardiovascular health post-event (e.g., after myocardial infarction or surgery), adherence rates among older adults remain low. At the same time, early incorporation of palliative care, centered on symptom relief and QoL, can support smoother transitions from acute care to hospice and improve overall patient well-being [7].

Moreover, advancements in technology and data collection are reshaping geriatric cardiology. Innovative tools such as smart watches, home blood pressure monitors, augmented/mixed reality (AR/MR) in interventional cardiology, and electronic patient-reported outcomes (ePROs) are gaining momentum [8,9]. However, their implementation raises questions about cost, ethics, and accessibility. Many older adults may lack the skills, confidence, or resources to effectively use these technologies, potentially exacerbating existing health inequities and limiting the benefits of digital innovation in vulnerable populations. To further refine diagnostic and treatment practices, adjusted cardiac biomarkers and enhanced data systems like the National Cardiovascular Data Registry (NCDR) and Practice Innovation and Clinical Excellence Registry (PINNACLE) are becoming increasingly vital [10-12]. These data registries help assess the effectiveness of various treatments and help improve the outcomes in cardiology therapy.

Ultimately, the study advocates for a holistic, interdisciplinary approach tailored to each individual. Shared decision-making, factoring in medical, psychological, and social dimensions, can significantly elevate patient satisfaction and care quality [13]. The significance of this review lies in its analysis of emerging evidence and expert consensus on age-adapted cardiovascular assessment, therapeutic adaptation, and care delivery. Key themes include individualized risk-benefit analysis, functional status assessment, and personalized treatment planning for conditions such as heart failure, atrial fibrillation, and ischemic heart disease in older adults [14].

In addition, it underscores the pivotal role of interdisciplinary collaboration in managing care transitions and addressing the social determinants of health, which disproportionately affect aging populations. By fostering collaboration among cardiologists, geriatricians, nurses, pharmacists, and social workers, healthcare systems can better meet the complex needs of this demographic [15].

Beyond its clinical implications, the review also seeks to influence research priorities such as those related to chemotoxicity or cardiac biomarkers, policy development, and a healthcare system design that focuses on a patient-centred approach. Its broader impact lies in promoting care models that improve clinical outcomes, support independence, reduce hospital readmissions, and ultimately enhance QoL for older adults [16]. Despite major advances in cardiology over the past decades, these improvements have not always translated into better outcomes for older adults, whose health profiles are often heterogeneous and marked by competing priorities [15,17].

Geriatric cardiology has emerged in response to these gaps, promoting a holistic, function-based perspective [18]. Contemporary research emphasizes the need to integrate frailty assessments, cognitive evaluations, and life expectancy considerations into clinical decision-making, factors traditionally overlooked in standard care models. Moreover, aggressive interventions may not always align with older patients’ end-of-life goals, further necessitating individualized and goal-concordant treatment plans [19].

This review extends these principles by synthesizing clinical evidence into actionable strategies for optimizing cardiovascular care in aging populations. It explores diagnostic challenges, therapy modifications, and the essential role of multidisciplinary care teams in maintaining independence and navigating transitions. Understanding that care delivery is varied and dependent on multiple factors, the review highlights the value of flexible, coordinated input from multiple specialties, offering system-level insights to foster healthcare environments that uphold autonomy and dignity in older age. [20].To that end, the study focuses on the delivery of cardiovascular care to older adults, particularly in the context of prevalent conditions such as heart failure, arrhythmias, and ischemic heart disease [3,17]. It includes updates on clinical guidelines, therapeutic innovations, and multidisciplinary care coordination [14,19]. Examples of both established practices and emerging areas of focus in geriatric cardiology are the discovery of cardiac biomarkers elevated with age, the implementation of a patient-centered approach, a review of chemotherapeutic drugs on cardiotoxicity, and much more.

This review also considers how physiological aging, frailty, and multimorbidity influence treatment outcomes. By drawing on literature from the past decade, it identifies both effective practices and persistent gaps in evidence, particularly for complex geriatric cases [20]. The ones dealing with a high cardiovascular burden. Let it be through toxicity or heart failure. Finally, it discusses how care delivery can shift toward being more person-centered and preventive, with the aim of fostering sustainable, equitable models for an aging world [21].

## Review

Approximately 54 million Americans are over the age of 65 years, a number predicted to cross 81 million by 2040. Around three-quarters of adults, 65-79 years of age, are suffering from CVDs, making it the leading cause of mortality [22]. However, since its diagnosis and management have advanced, the mortality rates in patients with CVDs have become comparable to those in the general population. Therefore, noncardiac etiologies are becoming more prevalent in the aged [23]. Endeavours to enhance general welfare in addition to life expectancy in the aged suffering from CVD are being promoted. 

Frailty, multimorbidity, and functional decline in cardiovascular aging

Frailty is a multidimensional syndrome marked by sarcopenia, weight loss, weakness, and reduced endurance. It is characterized by diminished physiological reserves and increased vulnerability to stressors, and is strongly associated with adverse cardiovascular outcomes, including heart failure, arrhythmias, and a heightened risk of mortality [17,24]. Frailty has emerged as an independent risk factor for CVD. In various studies, frailty has been associated with an increased risk of heart failure, myocardial infarction, stroke, and peripheral arterial disease. However, frailty is considered to have a bidirectional relationship with CVD [22]. The theory of ‘Post-hospital syndrome’ posits that a combination of sleep deprivation, immobilization, and disorientation leads to losses in various function domains (physical, psychological, social, etc.), rapidly increasing the rate of frailty and ultimately bringing about a higher rate of readmissions and mortality. It is of paramount importance to identify and manage frailty as it is a reversible and dynamic condition. Assessing frailty or pre-frailty using validated tools such as the Frailty Index, Fried’s Phenotype, or Essential Frailty Toolset can inform therapeutic decisions, emphasizing personalized care strategies [25]. 

Frailty not only worsens cardiovascular outcomes but also predisposes them to other chronic illnesses. Comorbidities compound with aging, causing chronic inflammation and insufficient tissue repair, further worsening CVD. Thus, multimorbidity, the coexistence of multiple chronic conditions, is highly prevalent in older adults with CVDs. Conditions such as diabetes, chronic kidney disease, and cognitive impairment interact with cardiovascular pathology, complicating treatment regimens [26]. Most of the current care models are single-disease specific and do not acknowledge discordant conditions, despite their impact on the overall outcome of patient satisfaction. However, the implementation of patient-centered care involves targeting treatment based on the diverse needs of the older population, leading to increased quality of life, rather than prolonging life with reduced functionality. This shifts the focus to the patient’s preferences, giving them a chance in decision-making. The array of multimorbidity requires adaptation of contemporary guidelines and incorporation of goal-oriented care [14,23]. 

Comorbidities cause limitations in activities of daily living and steepen the rate of physical and mental debilitation. Declining functional capacity in elderly cardiac patients leads to reduced independence and worsened prognosis. Impaired mobility, fatigue, and cognitive decline can limit adherence to treatments and rehabilitation efforts. There is a considerable lack of knowledge regarding the prevalence, assessment, and management of cognitive impairment in older adults with CVDs. The failing heart affects the cerebral function, while impaired neuronal signals can impact the heart, leading to a reciprocal association, defined as a “cardiocerebral syndrome” by Gorodeski et al. Studies have shown increased rates of 30-day hospital readmissions in heart failure patients with cognitive decline [5]. Exercise-based interventions, cardiac rehabilitation, and lifestyle modifications have shown promise in preserving functional and cognitive status [27]. Tailoring interventions to the patient’s functional level, including screening for cognitive decline, can optimize outcomes and maintain independence [28,29].

**Figure 1:**
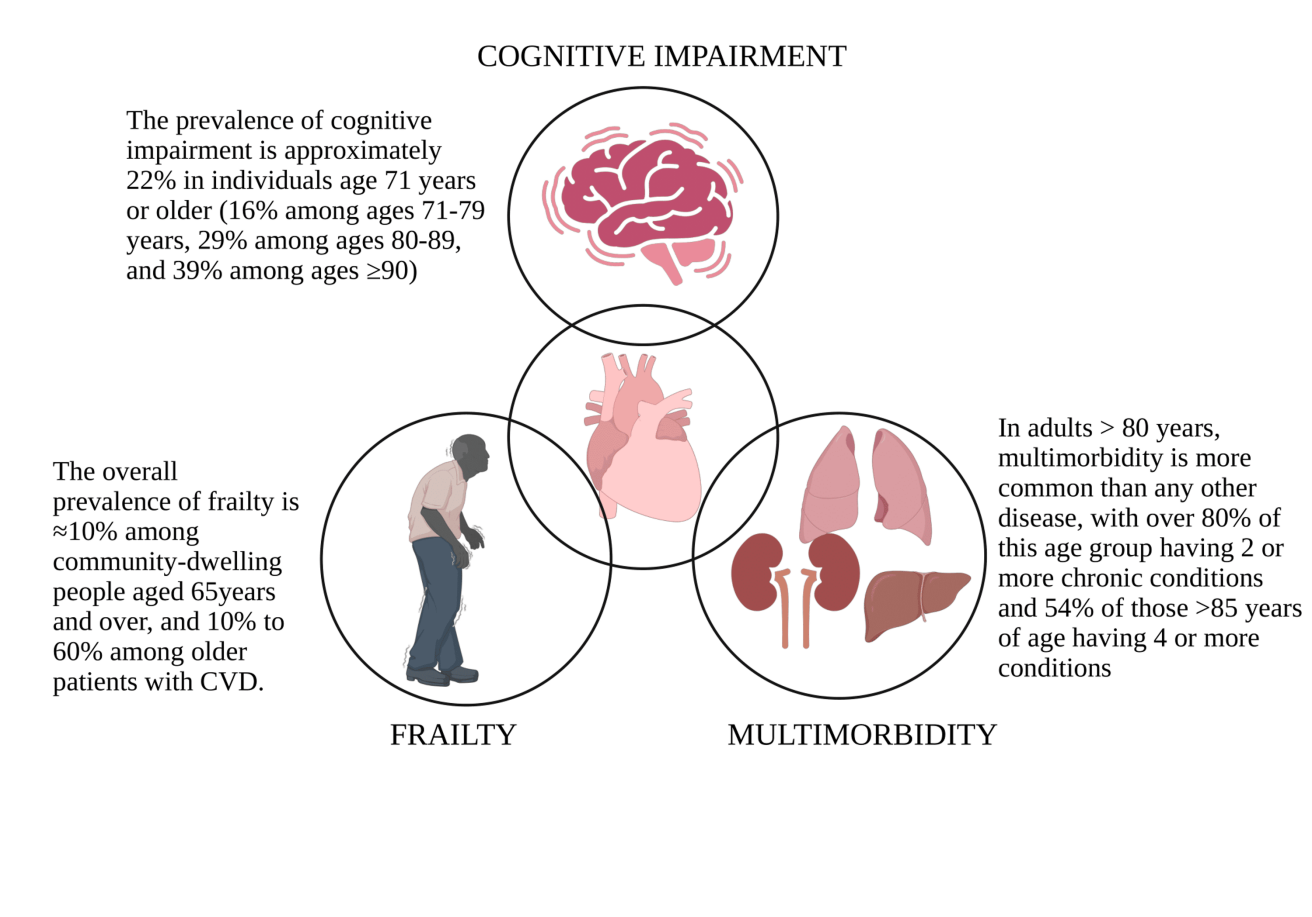
Prevalence of frailty, multimorbidity, and cognitive impairment in geriatric patients with cardiovascular diseases, signifying that they are interconnected Data adapted from [5,14,23] to create an image. Image credit: Shreya Agarwal

Frailty and multimorbidity both have a complex interplay with the cardiovascular system, leading to accelerated functional decline in the elderly, as shown in Figure 1. Understanding and addressing these interconnected conditions is essential for optimizing care in geriatric cardiology. Multimorbidity is also a key factor of polypharmacy [4]. Although often necessary, polypharmacy introduces risks of adverse drug reactions and treatment burden. All of these aspects, when combined, are detrimental if not intercepted expeditiously. Older patients lean towards improved function over an extended life with decreased activity. Integrating geriatric assessments into routine cardiology practice, promoting shared decision-making, and incorporating palliative approaches when appropriate strikes a balance between the standardised care and individualised care, centering on the requisites of the patient. According to the 2023 American Heart Association (AHA) Guideline for Management of Patients with Chronic Coronary Disease, a patient-centered approach ensures effective communication to optimize outcomes and improve the QoL of the patient. Geriatricians, who are acquainted with the patient’s goals of care, should be an integral part of this team [5], as they provide a holistic approach.

Diagnostic and therapeutic challenges in the aging cardiovascular system

**Figure 2:**
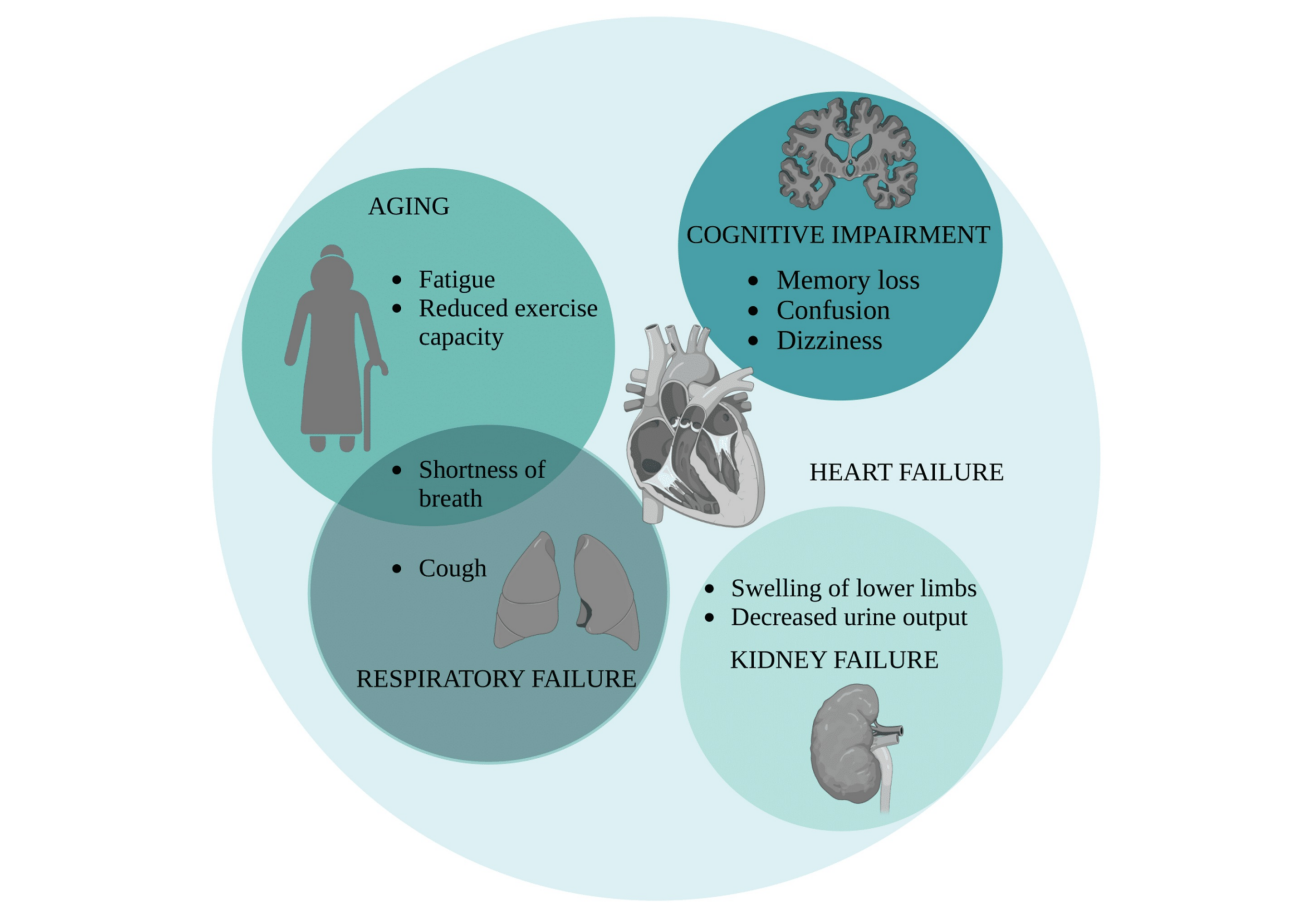
Figure demonstrating how different multimorbidities have similar symptoms to those of heart failure, leading to a delay in diagnosis or misdiagnosis Image credit: Shreya Agarwal

As people live longer, CVDs are becoming more common in older adults, but managing them is not straightforward. The textbook “chest pain” is usually absent in women, diabetics, and the elderly. Age-related structural and functional changes in the cardiovascular system lead to unique diagnostic and therapeutic challenges. These complexities arise due to overlapping comorbidities, atypical clinical presentations, and altered physiological responses to treatment [14]. Aging brings changes to the heart and blood vessels, like stiffer arteries and a weaker heart muscle, which can mask or alter the usual signs of disease. Older adults often exhibit nonspecific symptoms such as fatigue, breathlessness, or confusion instead of classic chest pain in conditions like myocardial infarction, making early recognition difficult. As a result, conditions like heart failure or arrhythmias may be harder to recognize, especially when symptoms are vague or overlap with normal aging [3]. Older patients also tend to have other health issues like diabetes, kidney problems, or memory loss. As illustrated in Figure 2, the presence of multiple chronic conditions such as long-standing diabetes mellitus, hypertension, and their complications can obscure cardiovascular symptoms or complicate diagnostic interpretation.

Medications that help one condition might worsen another or interact with other drugs, especially when many medicines are prescribed together, a situation called polypharmacy, which is the long-term use of five or more medications. With a prevalence of 39% in 2012, polypharmacy leads to worsening cognition, deterioration of nutrition, and increased financial burden [30]. This also leads to various adverse drug reactions and prescribing cascades. Nonsteroidal anti-inflammatory drugs (NSAIDs), given for pain due to a chronic disease, may worsen underlying cardiovascular conditions and increase blood pressure [31]. Calcium channel blockers (CCB), therapeutic for hypertension, can further exacerbate heart failure. Amlodipine, a CCB, can lead to lower extremity edema. If the edema is recognised as an independent symptom of heart failure and not a side effect, the addition of diuretics instead of deprescribing may lead to hypokalemia [32]. Pharmacokinetic and pharmacodynamic changes due to hepatic and renal function decline necessitate careful dose adjustments to prevent toxicity [33]. 

Another challenge is that most clinical guidelines are based on studies in younger people. Older adults, especially those with multiple health problems, are often left out of clinical trials [34]. That means doctors are sometimes left guessing whether a treatment proven in younger patients will work the same way in a 70-year-old with frailty and other conditions. Echocardiographic assessment may be challenging due to poor acoustic windows in frail elderly individuals. Changes like increased left ventricular wall thickness and valvular calcifications, which may occur with age, resemble changes due to heart failure, making it difficult to differentiate. There is a lack of a defined threshold beyond which these structural changes, which may be seen with aging, should be considered pathological in echo. Similarly, biomarkers such as troponins may be chronically elevated with age, requiring careful interpretation. Troponin elevation may be a result of declining renal function and altered build that develops with aging [35]. Hormonal changes that occur in elderly and post-menopausal women also affect troponin and Brain Natriuretic Peptide (BNP). These factors need to be taken into account. Further research is required in this area to establish adjusted levels of BNP for older adults to prevent misdiagnosis.

Even when effective treatments exist, like cardiac rehabilitation, they are not always used in older adults due to barriers like poor mobility or lack of support. Procedures such as valve replacements or stenting may carry more risks for frail patients, so these decisions need to reflect the individual's values and goals [36]. Frailty, cognitive decline, and reduced physiological reserve heighten perioperative risks for procedures like coronary artery bypass grafting (CABG) or transcatheter aortic valve replacement (TAVR) [28]. Digital health solutions, including telemedicine and remote monitoring of cardiac devices, are improving access to care and reducing hospitalizations. Anti-obesity medications and precision medicine, like genetic and biomarker-based approaches, are demonstrating improved cardiovascular benefits. New pharmacological agents such as sodium-glucose cotransporter 2 (SGLT2) inhibitors and glucagon-like peptide-1 (GLP-1) receptor agonists are showing promise in managing heart failure with preserved ejection fraction (HFpEF) in elderly patients [37]. To move forward, we need better tools to assess frailty and tailor treatments accordingly. 

The balance between aggressive intervention and quality-of-life considerations becomes crucial, requiring multidisciplinary collaboration and personalized treatment approaches [38]. More inclusive research is essential to create guidelines that reflect the real-world challenges of caring for older adults with heart disease. A team-based, patient-centered approach can help navigate these complexities and improve outcomes for this growing population. Given these diagnostic and therapeutic obstacles, optimizing cardiovascular care in older adults demands a comprehensive, individualized strategy that integrates geriatric principles with cardiology expertise. Future advancements in precision medicine and innovative imaging techniques may help mitigate these challenges, improving outcomes for aging populations.

Comparative effectiveness of comprehensive geriatric cardiology versus traditional disease-focused models

CVDs remain the leading cause of morbidity and mortality worldwide, and this burden is especially pronounced in the elderly. However, the way older adults present and respond to treatment often differs markedly from that of younger patients, posing challenges to traditional disease-focused care models. Conventional cardiology tends to rely on guideline-directed therapy aimed at singular pathologies, but this approach frequently falls short in addressing the nuanced and overlapping issues faced by older adults, namely, frailty, multimorbidity, polypharmacy, and cognitive impairment. Due to this, clinicians encounter the dilemma of treating complex cardiac diseases in the aged population. The burning question for modern cardiology is not just how to treat the disease, but how to treat the patient [14].

In routine care, elderly CVD patients rarely present with isolated cardiac issues. A typical example might be an 80-something-year-old with heart failure, mild cognitive impairment, and arthritis, recently hospitalized due to medication non-adherence. Under traditional care, treatment may center on optimizing ejection fraction and titrating medications, often guideline-driven. However, comprehensive geriatric cardiology (CGC) offers a more holistic, integrated care delivery model that extends beyond initial diagnosis. CGC is not merely a diagnostic approach using comprehensive geriatric assessment (CGA), a structured, multidimensional evaluation of an older person’s medical, functional, cognitive, and psychosocial health; it is a longitudinal, multidisciplinary, person-centered framework. It addresses the full complexity of aging patients by evaluating cognitive function, functional status, fall risk, and social context; simplifying medications; and aligning care with patient goals. This often involves prioritizing symptom control and functional independence over aggressive disease optimization [13].

**Figure 3:**
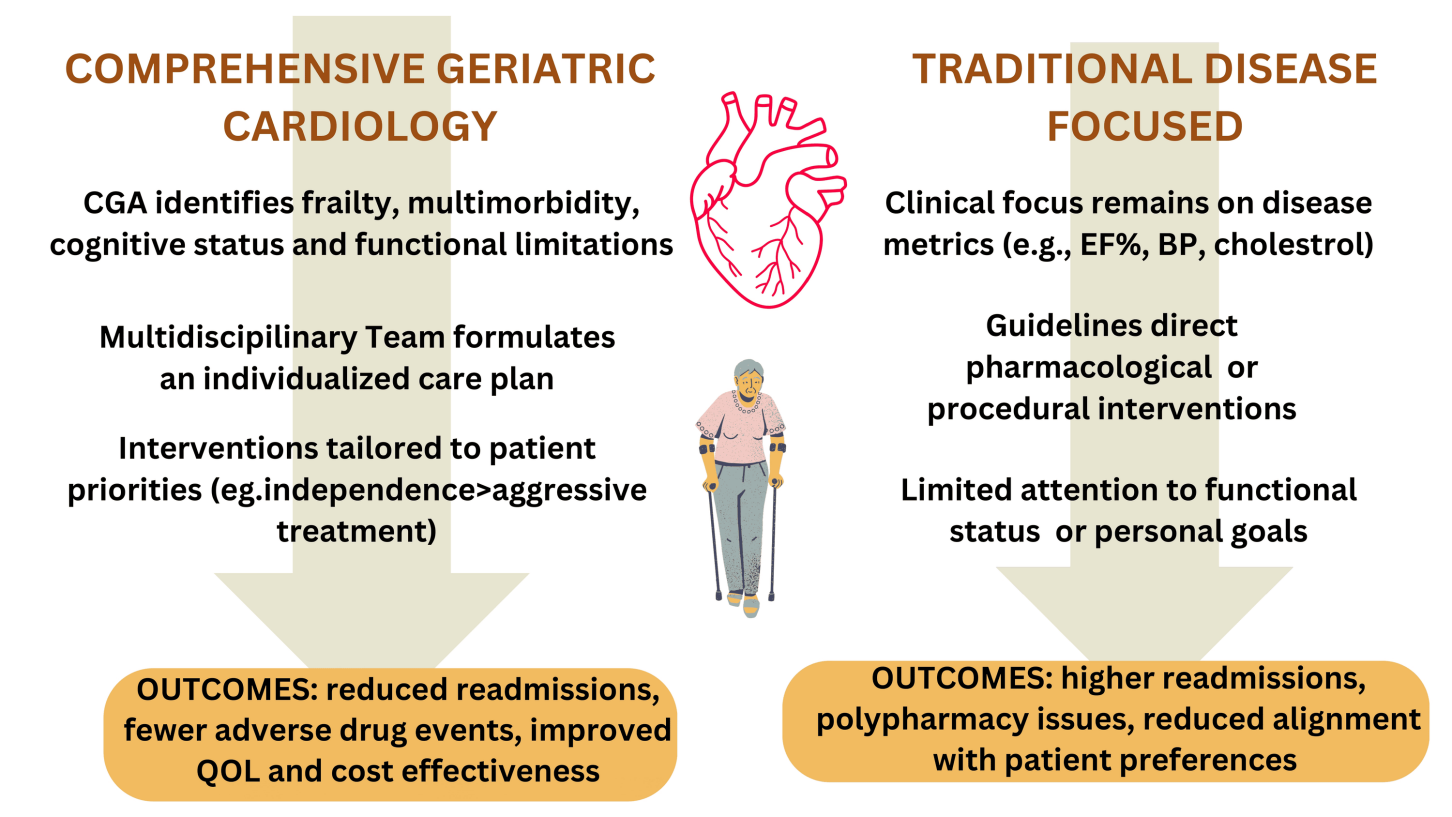
Image comparing the effectiveness of comprehensive geriatric assessment with that of the traditional disease-focused approach Image credit: Siri Chithanuru

CGC offers a multidimensional model that integrates geriatric principles into cardiovascular management by recognizing that elderly patients often prioritize autonomy, cognitive preservation, and functional independence over aggressive disease-specific interventions. Within the CGC framework, tools like the CGA are used not only to identify deficits but to guide interdisciplinary care planning based on a patient’s broader health context and goals. CGA thus forms the foundation of a more coordinated, person-centered care strategy. Figure 3 illustrates how CGC diverges from traditional cardiology models, contrasting their respective approaches to assessment domains, care planning processes, and outcome priorities.

The effectiveness of CGC is supported by a growing body of literature, which has been outlined in Table 1. For instance, in an RCT, Ekerstad et al. (2016) and Rodriguez-Pascual et al. (2014) found that frail elderly patients treated in an acute care setting had significantly reduced three-month mortality, fewer rehospitalizations, and better QoL when CGA-based interventions were applied to elderly CVD patients [39,40]. Systematic reviews, including that of Ellis et al. (2017), which analyzed 29 RCTs involving over 13,000 patients, concluded that hospitalized older adults who received CGA were more likely to return home independently and had lower institutionalization rates [41]. Even where evidence is graded low in quality, such as in Raijmann et al. (2025), it still offers valuable insights, especially given the ethical and practical barriers in conducting randomized trials among frail elderly populations. In such cases, real-world data, observational studies, and expert consensus become essential complements to traditional evidence hierarchies. However, CGC remains underutilized due to practical challenges, including time constraints, a lack of geriatric training among cardiologists, and poor coordination between specialties.

**Table 1:** Effectiveness of comprehensive geriatric assessment RCT: randomised controlled trial, CGA: comprehensive geriatric assessment

Authors, year	Sample	Methodology	Key findings
1. Ekerstad N, et al. (2016) [39]	408 frail elderly patients in Sweden	Randomized controlled trial comparing acute CGA unit care vs. conventional care	CGA unit care led to lower three-month mortality (HR = 0.55), improved quality of life, and fewer rehospitalizations.
2.Rodríguez-Pascual, et al. (2014) [40]	487 patients ≥75 with decompensated heart failure	Prospective cohort study assessing CGA scores at discharge	Higher CGA scores are linked to increased two-year mortality in older patients hospitalized for heart failure in an acute geriatric unit.
3.Ellis, et al. (2017) [41]	29 RCTs with 13,766 participants	Cochrane systematic review	CGA improved the odds of being alive and at home at follow-up vs. usual care.
4. Raijmann R, et al (2025) [42]	Various studies on older cardiac patients	Systematic review	Low-quality evidence of lower complications and better quality of life with CGA.

Moreover, CGC promotes interdisciplinary collaboration, drawing on the expertise of geriatricians, cardiologists, nurses, pharmacists, and social workers. This team-based approach ensures that care is not only medically appropriate but also socially and functionally sustainable. By contrast, traditional cardiology care often adheres strictly to disease-specific guidelines, with limited screening for geriatric syndromes and little integration of patient preferences or functional goals. Such disease-focused care can lead to fragmented management, redundant interventions, and poor recognition of geriatric syndromes such as delirium, falls, or incontinence-conditions that significantly influence outcomes but often remain undiagnosed in standard models [42]. These prognostic evaluations commonly ignore the most critical spider in the web of older people's prognosis (the cognitive and functional status), that are the heart of any CGA intervention. However, if implemented rigidly, CGA can become overly checklist-driven, potentially crowding out relational care elements such as empathy, shared decision-making, and trust. To counteract this, CGC should emphasize goal-aligned, narrative-based care planning that ensures patients and caregivers remain active partners in care decisions.

Despite its proven advantages, CGC is still underutilized. Barriers include limited geriatric training among cardiovascular specialists, insufficient time for comprehensive evaluations, and poor integration between specialties [41]. However, the limitations extend beyond these factors. The cost-effectiveness of CGC programs remains a concern, particularly in resource-constrained healthcare systems with time constraints, due to barriers in the translation of guidelines into clinical settings and a lack of familiarity. However, emerging evidence suggests CGC may reduce long-term costs through lower readmission rates, improved medication adherence, and fewer hospital-acquired complications. Nonetheless, further robust health economic evaluations in cardiology-specific populations are needed.

The heterogeneity of the elderly population, with varying degrees of comorbidities, functional status, and social support, makes standardized implementation challenging. Patients and caregivers value relational aspects of care, which may be undermined by a task-focused CGA approach. This can limit their involvement in decision-making and overlook their own capabilities and care strategies, especially after discharge [43].

There are very few clinical trials, with no proper evidence of the benefits of CGA implementation in acute settings. However, this lack of evidence reflects not ineffectiveness but the challenges of feasibility and safety in high-pressure environments such as emergency units, where time constraints, limited staffing, and rapid decision-making often preclude comprehensive assessments. Policy changes, medical education reform, and structural incentives may help overcome these obstacles. For instance, incorporating CGC principles into cardiology fellowships, offering team-based care incentives, and funding interdisciplinary training modules can bridge the divide between geriatrics and cardiology. In high-pressure or resource-limited settings, practical approaches such as phased implementation, tele-assessment tools, and embedding geriatric-trained nurses within cardiology teams may facilitate CGC integration. Additionally, future research should continue to evaluate the long-term benefits of CGC, especially in diverse healthcare settings, to validate its scalability, sustainability, and impact on healthcare utilization. Addressing these challenges through policy support, education, and inclusive research is essential to fully realize the potential of CGC.

As the global population continues to age, there is a growing need to complement traditional disease-centric models with more holistic approaches such as CGC. While many advanced cardiology programs already incorporate multidisciplinary care and recognize age-related issues, traditional models may still overlook important geriatric syndromes and patient-centered priorities. CGC frameworks offer a more structured, individualized approach that aims not only to improve clinical outcomes but also to preserve function and support what matters most to older patients - dignity, autonomy, and quality of life. Transitioning from traditional to CGC models is not only appropriate but necessary to meet the evolving needs of an aging population.

Enhancing QoL through multidisciplinary cardiovascular intervention

**Figure 4:**
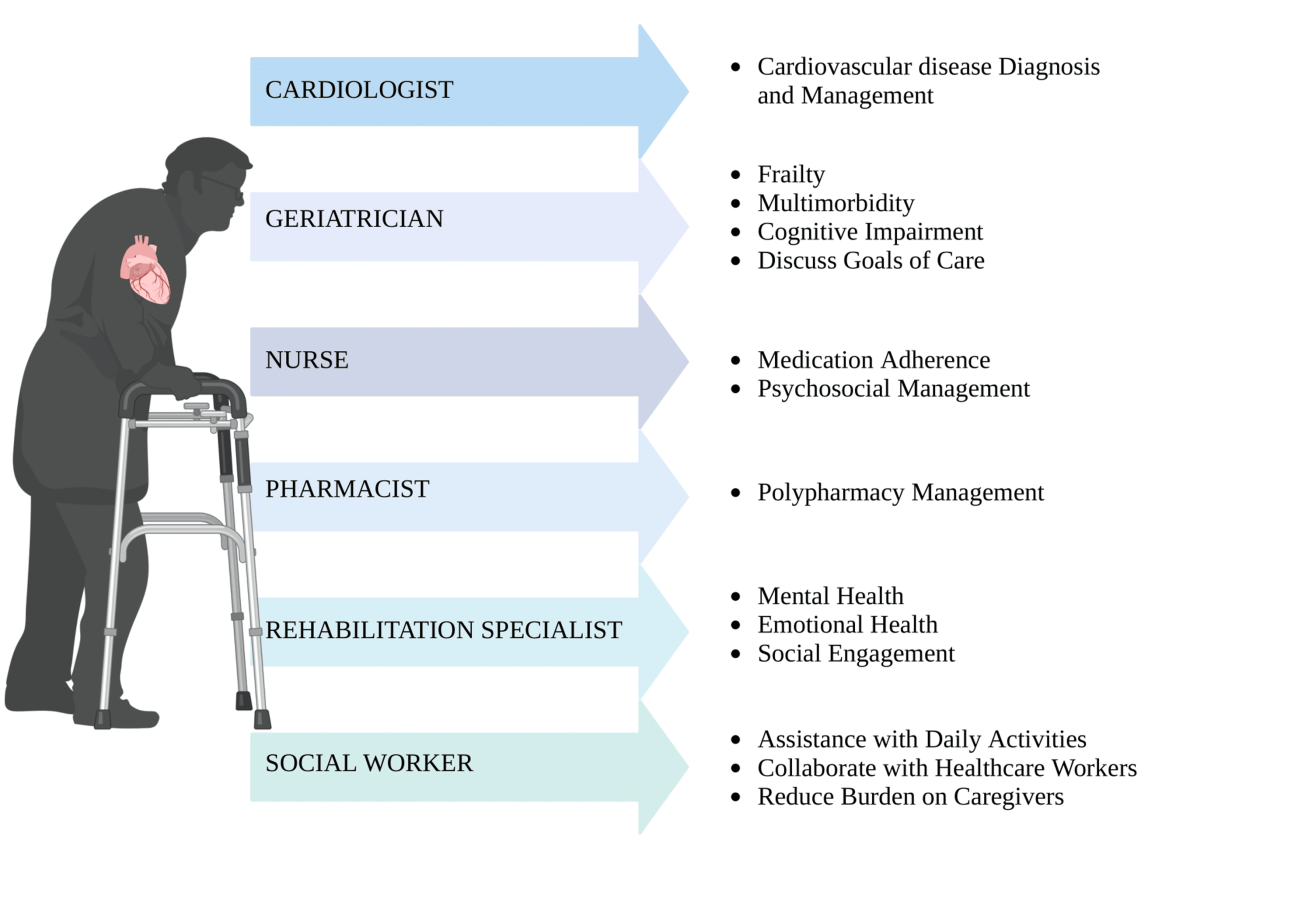
Roles of different healthcare workers in a multidisciplinary approach Image credit: Shreya Agarwal

Improving the QoL in geriatric adults with CVDs is not limited to cardiovascular treatment but to other factors, including frailty, functional status, emotional health, and social well-being. Multidisciplinary care approaches consisting of cardiologists, geriatricians, nurses, pharmacists, rehabilitation specialists, and social workers are broadly recognized as a holistic approach in optimizing cardiovascular care in the geriatric population (Figure 4).

A large retrospective cohort study involving over 3,200 patients with heart failure found that participating in outpatient multidisciplinary cardiac rehabilitation demonstrates a marked reduction in mortality, heart failure, and rehospitalization in one year, particularly older adults with frailty and functional limitations [44]. These programs address not only medical management but also structured care support, physical reconditioning, diet, and psychosocial counseling to improve life expectancy and reduce the physical, financial, and emotional burden of frequent hospital admissions. 

The SENECOR RCT displayed the inclusion of both a geriatrician and a cardiologist in the follow-up care after heart failure hospitalization led to a 33% reduction in all-cause hospitalization at one year in comparison to cardiologist-only care [45]. The geriatrician's follow-up care addressing concerns of multimorbidities and frailty and personalized goals reflected an impact on the multidimensional nature of Qol among the aging population. In a multidisciplinary approach, each member of the team provides a unique level of expertise, allowing for comprehensive management. Studies show that CGA enhances independence, reduces inappropriate medication, and supports personalized decision making, which are all linked to QoL [46]. 

Personalizing cardiac rehabilitation programs for older adults plays a key role in improving emotional health and reducing depression by increasing social engagement [47]. These programs recommend group-based physical activity, motivational counseling, and education that aligns with the patient’s capacity and priorities, helping them to regain confidence and autonomy. 

Caregiver well-being can indirectly improve the QoL. A recent review study assessing elderly cardiac patients with multimorbidity often experiences poor QoL, which contributes to the high burnout rates among their caregivers. A structured care that includes caregiver support, home services, and case management can reduce this burden and promote more stability among home environments [48].

Collaboration among team members promotes a smooth transition from diagnosis to long-term treatment. By valuing the patient’s preferences, a team-driven approach ensures long-term patient compliance and takes social determinants of health into account [2]. These may include factors such as housing instability, food insecurity, and lack of transportation, which can directly affect treatment adherence and recovery in elderly patients. This also enhances utilization of healthcare resources, especially in rural and underserved areas, where telemedicine, nurse-led clinics, and community health workers can help extend care and address gaps due to workforce shortages or limited specialist availability.

When nurse practitioners and general physicians conduct routine follow-ups, it reduces the burden on cardiologists and provides timely and cost-effective delivery of care. However, structural and logistical barriers such as reimbursement limitations and staff shortages can impede the full implementation of such multidisciplinary models.

Overall, a multidisciplinary cardiovascular approach enhances survival in older adults not only by reducing hospitalization and mortality, but also by improving mental health and maintaining functional capacity, leading to an improvement in quality of daily living. These approaches reflect a shift from a disease-centric model to one that is tailored to the individual, supporting a meaningful life. In addition, by reducing emergency visits and hospital readmissions, multidisciplinary care has the potential to generate substantial cost savings, although further health economic evaluations are warranted to fully understand its long-term value.

Addressing health inequities and disparities in cardiogeriatric populations

**Figure 5:**
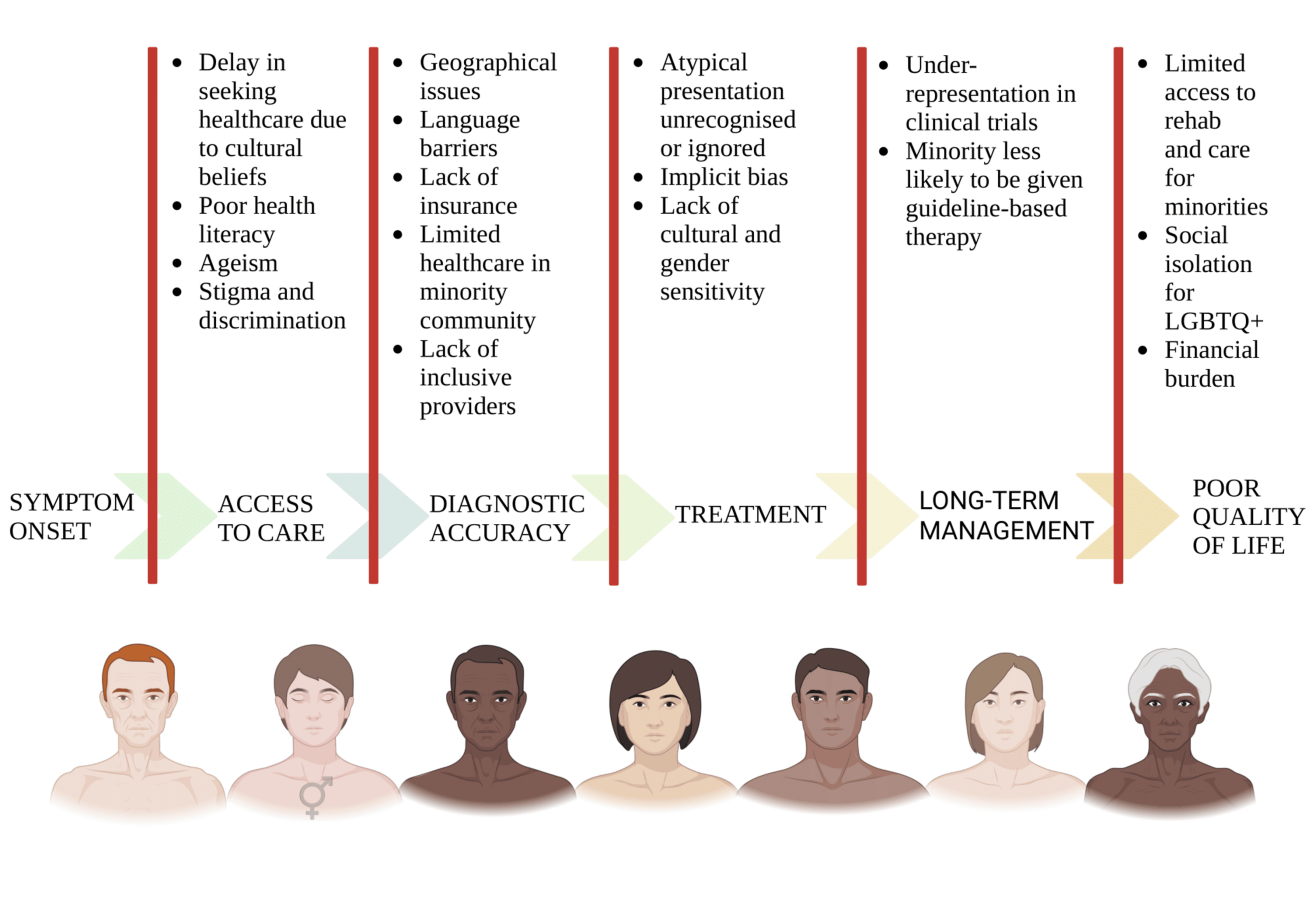
Various barriers faced by the minorities and the LGBTQ+ communities in each step from onset to symptoms to long-term management Image credit: Shreya Agarwal

This narrative review focuses on the large and persistent health disparities that the elderly population with CVDs faces across a variety of demographic, socioeconomic, and geographical situations (Figure 5). Our findings highlight that racial and ethnic minorities, those with lower socioeconomic status, individuals with limited health literacy, and older adults living in rural or underserved areas face disproportionately high disparities in access to care, diagnostic accuracy, treatment efficacy, and health outcomes. 

In a study conducted by Mathews et al., involving 728 participants of the ARIC (Atherosclerosis Risk In Communities) study with acute decompensated HFrEF (heart failure with reduced ejection fraction), people belonging to lower economic section and of lower education had increased rates of mortality and readmission (with hazard ratios of 1.52 and 1.45, respectively) [49]. According to Javed et al., Black adults experience a higher burden of CV risk factors such as hypertension and obesity and are more than twice as likely to die of CVDs, relative to White adults. Similarly, American Indian individuals are 1.5 times as likely to be diagnosed with coronary heart disease, compared with the White population [50].

These findings highlight a systemic issue in which aging and CVD interact with social determinants of health, posing additional challenges for vulnerable people. Despite advances in cardiovascular medicine, many older persons continue to have limited access to evidence-based medicines and preventative care as a result of structural and institutional disparities. Structural racism and ageism manifest in forms such as biased risk assessment models and inadequate physician-patient communication. This inequitable landscape has a profound impact on healthcare systems, increasing hospitalisations, procedure difficulties, and long-term care needs.

Our findings are consistent with a growing body of data that shows ageism, socioeconomic adversity, and racial inequality as major causes of poor cardiovascular outcomes in older persons [51]. According to studies, minority elderly people are less likely to obtain guideline-directed therapy for illnesses, including heart failure and myocardial infarction, and they are more likely to experience care delays. These disparities are further increased for those who occupy intersecting identities, such as being both elderly and LGBTQ+, or elderly and a racial minority, amplifying barriers to trust, culturally competent communication, and continuity of care. Furthermore, the under-representation of older persons in clinical trials limits the applicability of new treatments to this population, reinforcing clinical ambiguity and heterogeneity in care. This under-representation is possibly due to strict inclusion criteria, provider bias, and concerns about polypharmacy. Strategies such as inclusive eligibility criteria and community engagement are essential to close this gap.

The importance of addressing these inequities stems from the potential to significantly improve health outcomes for one of the fastest-growing patient populations. Healthcare providers, researchers, and policymakers can build a more inclusive and effective healthcare environment by recognising and actively addressing the social and systemic barriers to unbiased cardiogeriatric care. Approaches to be considered include incorporating social determinants of health (SDOH) screening into clinical workflows, expanding telehealth infrastructure in rural areas, and incentivizing equity-focused research initiatives. Impartial treatment models, culturally sensitive patient education, and targeted policy reforms have the ability to close disparities and guarantee that advances in cardiology benefit all older persons, not just those who have access or privilege.

As a narrative review, our work is naturally constrained by selection bias and the lack of a systematic review approach. The heterogeneity in study designs, populations, and healthcare systems throughout the examined literature further limits the capacity to generalize findings globally. Furthermore, some areas, such as inequities among LGBTQ+ older persons with CVDs, remain under-researched, indicating a larger gap in the literature. Using Behavioural Risk Factor Surveillance System data from 35 US states, researchers have found that LGBTQ+ Americans have higher rates of poverty (21.6%) than their counterparts (15.9%), with especially high poverty rates among transgender people (29.4%) and bisexual cisgender women (29.4%) [52].

These findings must be examined within the context of global population ageing, the increased prevalence of chronic CVDs, and growing recognition of health equity as a public health priority. Efforts to improve cardiogeriatric care must be integrated into larger efforts towards value-based care, community health engagement, and cross-sector collaboration. To address gaps, systemic reforms in healthcare access, education, and legislation will be required, in addition to clinical innovations.

Ultimately, addressing health disparities in cardiogeriatric populations requires not just clinical efficacy but also moral and social responsibility. As we strive toward a more reasonable healthcare future, cardiovascular care reform, research, and policy must prioritise the needs of our ageing populations, particularly those who are most vulnerable.

Innovations and evidence-based models in geriatric cardiology

The main challenges in managing cardiac conditions in geriatric cardiology and in which modern medicine is looking for innovations and updates, are multi-morbidity, frailty, and polypharmacy. In this subheading, we will look at evidence from recent publications and some of the practical ways to overcome challenges in each category.

Multimorbidity (Presence of Two or More Chronic Conditions in >70% of Older Adults)

Although older adults have multiple coexisting illnesses, the trend still continues in treating them in an individual disease-based approach rather than providing holistic care. Team-based care is foundational in Geriatric care. Advanced practice nurses, physicians assistants, pharmacists, nutritionists, physical and occupational therapists, social workers, palliative care and hospice consultants should be involved in managing multimorbid conditions together. Most importantly, geriatric cardiologists have to be involved in prioritising treatments and integration of care from each specialist, thus avoiding fragmentation of care. Palliative care specialists should work along with Geriatric cardiologists in the later stages of diseases if the goal of the patient is to relieve pain and improve symptoms. The Geriatric 5Ms (Mind, Mobility, Medications, Multi-complexity, and What Matters Most) provide a useful lens for understanding the multidimensional needs of older adults. These principles can help inform holistic care approaches, especially when integrated with disease-specific assessments and interventions [53]. Such team-based care requires strong communication skills to coordinate care among different specialties and to tailor management according to goals and management of the patients and their caregivers. Therefore, effective communication should be practiced by physicians to manage geriatric patients who are undergoing treatments and under the care of various specialists [14,54].

Frailty (Loss of Physiological Reserve and Vulnerability to Stressors)

Cardiologists should screen for frailty in at-risk individuals, and it should be made a routine practice. Earlier, we identified frailty; there is a better chance of outcome, and the patient’s quality of life. Proper nutrition, cognitive training, and physical training are important factors to consider in managing and reversing frailty. Proper nutrition with adequate calorie intake and micronutrients is a crucial part of a multidisciplinary approach. Cognitive training involves memory, planning, reasoning, visuospatial skills, attention, switching tasks, arithmetic, verbal fluency, problem-solving, exergames, psychomotor exercises, and square stepping. In a systematic review that compared the outcomes of exercise and cognitive training given separately and collectively, it was found that the combination of both was associated with a better outcome. Also, patients who were trained in groups had better outcomes in all cognitive categories [55]. Aerobic and anaerobic exercises have their own benefits. Aerobic exercises should be advised with caution and only to patients who are not at risk of injurious falls. Furthermore, cardiac rehabilitation has been proven to improve mild to severe admission frailty. Therefore, the usage of cardiac rehabilitation services has to be increased [56].

Polypharmacy

Unwanted drug prescription in geriatric adults is one of the main reasons for morbidity and mortality in older adults. Richter et al., in a narrative review of frailty in cardiology, suggest using the Beers criteria in North America and the STOPP-START criteria in Europe as screening tools that must be used to prevent unwanted drug prescription in geriatric adults [57]. Cardiac drugs are continued for years without dosage reduction or deprescription; however, the efficacy of any cardiac medication has not been proven when taken for more than 3 years [38]. Hence, deprescription is important in preventing adverse drug reactions, morbidity, and mortality. Rationale and evidence in deprescribing common cardiac medications have been outlined in a narrative review by Karen Ho [58]. The summary is provided in Table 2.

**Table 2:** Deprescribing common cardiac medications CAC: coronary artery calcium, CVD: cardiovascular disease Note: Deprescribing statins in patients >75 years those who are taking statins from their younger age causes an increase in cardiovascular events. Thus, deprescription in such patients should be done with caution.

Drugs	Deprescription	Evidence
Statins	*Stop prescribing for primary prevention of CVD in >75 years old. *Stop prescribing if the CAC score is zero in 76-80 years.	*No current formal recommendations available. *The 2018 American Heart Association guideline gives a IIB recommendation [58].
Antihypertensives	*Stop prescription if the blood pressure is <140 mmHg in patients of Clinical frailty score >/=7. Target range is 140-160mmHg.	*Mallery et al. consensus guidelines [58].
Aspirin	*Stop prescribing in >70 years and those having bleeding disorders.	*The most-recent 2023 Canadian Cardiovascular Society guidelines for the use of antiplatelet therapy and The 2019 American College of Cardiology/American Heart Association guideline [58].

Future directions in geriatric cardiology: research, policy, and preventive strategies

As life expectancy continues to rise, physicians are encountering an increasing burden of CVDs in older adults. These patients frequently deal with multiple health conditions, making cardiovascular treatment harder. A geriatric cardiologist must look beyond just treating the heart and understand the whole person, including their goals for life, social situation, and physical limitations [59].

Aging changes how the body reacts to tests and treatments. Tests like troponin blood levels may be naturally higher in older people, even without a heart attack, leading to unnecessary treatments or missed diagnoses [10]. In addition, levels of natriuretic peptides, used in diagnosis for heart failure, rise even in healthy older adults due to declining kidney function and body composition [60]. Myeloperoxidase, a marker for oxidative stress, is linked to plaque instability and cardiovascular risk. It may help to identify older patients at an increased risk of heart attacks. Thus, scientists are calling for age-adjusted diagnostic tools and personalized guidelines for older adults, which would ensure that patients are not under- or over-treated. 

Many older adults deal with both cancer and heart disease. This is especially risky because cancer treatments can damage the heart and blood vessels. Anthracyclines cause a type I cardiotoxicity, leading to irreversible myocardial cell loss. Trastuzumab can cause a type II cardiotoxicity leading to reversible dysfunction at the cellular level, which may ultimately cause permanent damage if not intervened. Targeted therapies such as VEGF inhibitors and tyrosine kinase inhibitors can cause myocardial stress, hypertension, and endothelial dysfunction. Lastly, radiation therapy can generate reactive oxygen species, causing endothelial damage, fibrosis, and arteritis, manifesting years later in any area of the heart. Hence, doctors are working together to form cardio-oncology teams, which will develop a safe and balanced treatment plan, avoiding making one condition worse while treating the other [6].

Researchers are working on new treatments, going beyond the standard medications. Newer drugs, such as proprotein convertase subtilisin/kexin type 9 (PCSK9) inhibitors and SGLT2 inhibitors, have been successful in lowering cholesterol and improving outcomes [59]. These have fewer side effects and work better in older adults who are sensitive to traditional drugs. Novel oral anticoagulants (NOACs) like dabigatran and rivaroxaban offer safer, more convenient alternatives to warfarin. They allow for fixed dosing without monitoring and reduced drug interactions. Although these have advanced cardiovascular care, their broader use may be constrained by high costs, potential adherence difficulties, renal function limitations, and the limited inclusion of elderly patients in clinical trials. Future treatments may even include stem cell therapy or gene editing, which might one day help repair damaged heart tissue. However, more testing is needed to see how these treatments work in the elderly.

Technology is playing a bigger role. Devices like smartwatches, home blood pressure monitors, and wearable heart rate trackers allow doctors to check patients remotely. However, while these tools are becoming more accessible, many older adults face barriers such as low digital literacy, visual impairment, cognitive decline, or motor limitations that can hinder effective use. To ensure equitable access, healthcare providers and policy leaders must prioritize user-friendly design, caregiver involvement, and affordability. More funding for training programs and subsidized devices can help close this gap.

Patient-reported outcomes (PROs) are surveys or tools that include patient feedback. They provide information that doctors might miss in a short visit. Studies have shown that patients sharing their symptoms and goals leads to better treatment and improved quality of care. In a recent trial, patients using electronic PRO systems had better satisfaction and communication with their care teams. All patients in the electronic PRO group completed the surveys successfully, indicating the system was feasible and user-friendly among older adults [9].

Palliative care, focusing on making patients feel better physically and emotionally, should also be a bigger part of treating older adults. This helps manage pain, shortness of breath, and anxiety, and supports patients in decision making [61]. Even though this care is helpful, it’s underutilised [62]. Doctors should be trained to start these conversations earlier, so patients can plan ahead and get the support they need. Making choices about treatment, surgeries, heart devices, or strong medications can be difficult for older patients, as it may affect their independence. Shared decision-making means the doctor and patient work together to choose the best option based on the patient’s values [63].

**Figure 6:**
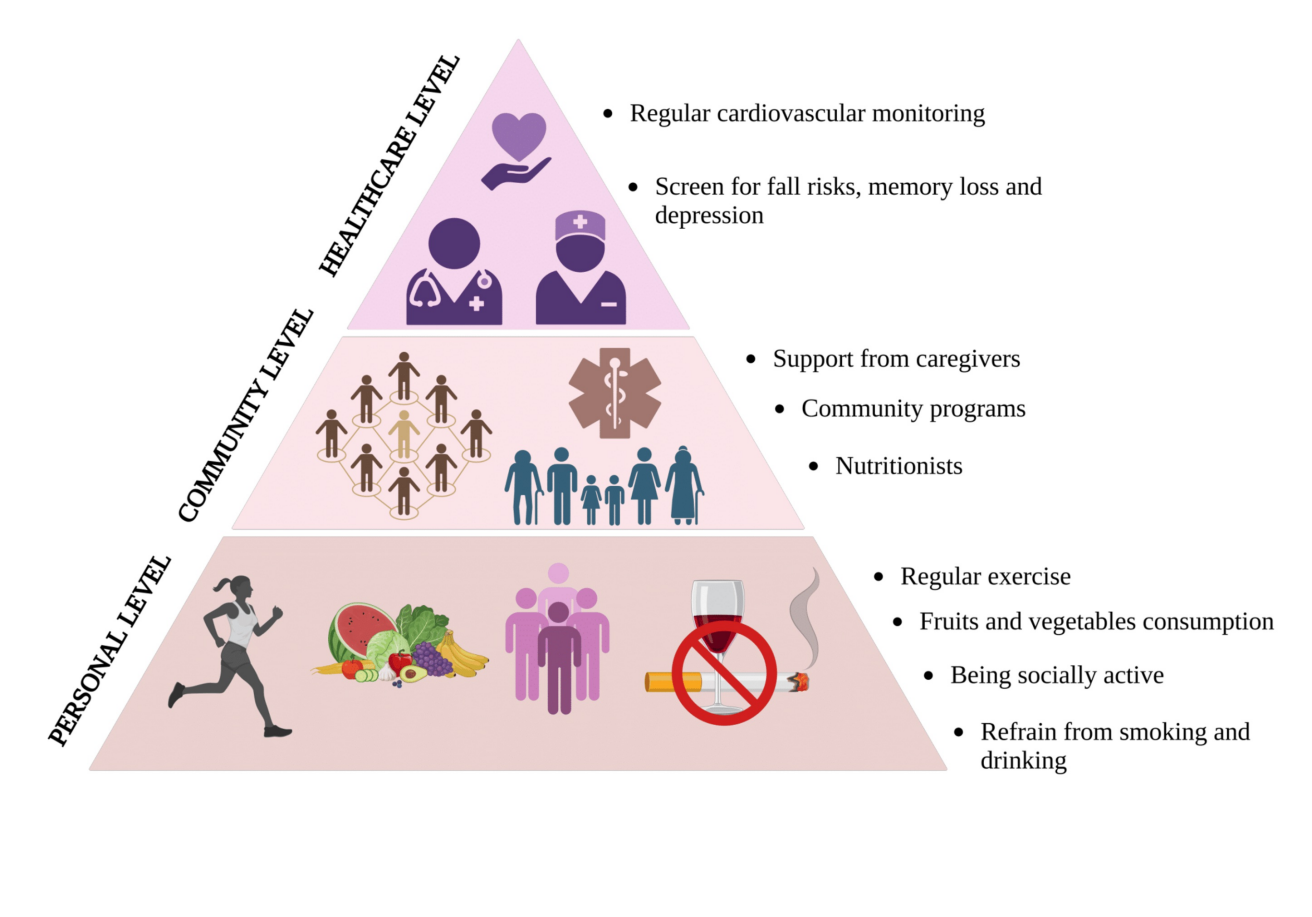
Image explaining how cardiovascular diseases in the elderly can be prevented at separate levels Image credit: Shreya Agarwal

Encouraging regular exercise, healthy eating, staying socially active, and avoiding smoking can help prevent heart disease and improve quality of life. For example, following the DASH diet to lower hypertension, participating in structured exercise programs like walking groups, and engaging in senior community initiatives such as group activities or volunteer opportunities have shown benefits in older adults. Doctors should also screen for fall risks, depression, and memory loss, as these impact heart health. Support from caregivers, community programs, and nutritionists helps older adults stay independent and out of the hospital. Figure 6 depicts the various methods to improve patient well-being.

In the future, we need more research that includes geriatric patients. Many clinical trials focus on younger adults, leaving us unaware of how treatments affect the aged. Medical schools and residency programs should teach caring for aging patients. Policy makers must support programs offering whole-person care and remove barriers, like cost and transportation. This can include expanding Medicare coverage for telehealth, offering incentives for geriatric specialization, and increasing funding for community-based health and wellness programs. By focusing on personalized and coordinated care, the field of geriatric cardiology can truly meet the needs of our aging population.

## Conclusions

This narrative review delves into the CVDs affecting a significant subset of the general population, the elderly. From presenting symptoms to diagnosis and its respective treatment, age has a remarkable impact on CVD. While in the younger population, the treatment guidelines aim for improvement of the disease, the elderly require a comprehensive approach that focuses on improving quality of life as well. We have pressed upon the need for a multidisciplinary model of care and its effectiveness in the mental and social aspects of life. Multimorbidity and polypharmacy are among various facets that need to be taken into account for the diagnosis and long-term management of any CVD.

One must also be aware of the various biases that are faced by the elderly of different economic, social, and ethnic statuses. These include ageism in clinical trials, disparities in access to specialty care, and underdiagnosis in marginalized subgroups, including women, minorities, and LGBTQ+ older adults. A treatment model should be created that is inclusive of different ages, races, and sexualities.

These key challenges need to be faced with age-adjusted diagnostics, including the use of tailored cardiac biomarker thresholds (e.g., troponin, NT-proBNP), imaging criteria, and functional assessments specific to older populations. Safer medications such as NOACs and PCSK9 inhibitors, collaboration between teams, and the integration of technology and palliative care are also essential. Preventive strategies such as lifestyle modifications, regular screening, and community-based support should be prioritized as part of comprehensive geriatric cardiovascular care.

Due to the dearth of research, we call for inclusive studies and improved training to deliver patient-centered care. Addressing these challenges requires curricular reform guided by geriatric care frameworks to better prepare healthcare professionals for the complexities of geriatric cardiovascular medicine.
